# Supplementary material for: Using Real-World Data to Determine Health System Costs of Ontario Women Screened for Breast Cancer
Source: Curr Oncol. 2022 Oct 31;29(11):8330–9. doi: 10.3390/curroncol29110657 (PMC9689006; doi:10.3390/curroncol29110657)
Supplement: Supplementary file 1 [file curroncol-29-00657-s001.zip › curroncol-1948164-supplementary.pdf]

## SUPPLEMENTARY MATERIAL

Table S1: Exclusion codes ........ 2

Table S2: Ontario specific diagnostic codes and unit costs ........ 2

Figure S1: Breast cancer screening schematic ........ 3

Table S3: Ontario specific cost assumptions ........ 4

Table S4: Quebec specific unit costs ........ 4

**Table S1.** Exclusion codes.

| OHIP FEE CODE | DESCRIPTION |
|---|---|
| R112 | Augmentation mammoplasty – Unilateral Implant |
| Z142 | Removal of Breast Prosthesis |
| E505 | Breast- with limited axillary node sampling |
| E506 | With Axillary sentinel node biopsy- add to R111 |
| E546 | With axillary node dissection up to axillary vein- add to R111 |
| R108 | Simple Mastectomy |
| R109 | Mastectomy –modified or radical modified (with or without biopsy) |
| R117 | Subcutaneous with nipple preservation |

**Table S2.** Ontario specific diagnostic codes and unit costs.

| CODE | DESCRIPTION | H (tech) | P (prof) |
|---|---|---|---|
| X172 | Mammogram (screening) – unilateral | $28.05 | $16.90 |
| X178 | Mammogram (screening) – bilateral | $37.15 | $27.00 |
| X194[a] | Additional coned views with or without magnification (limit of two per breast) per film | $5.95 | $5.20 |
| X166[b] | Examination using portable machine ($72.20 fee from Schedule of Facility Fees) | n/a | n/a |
| X184 | Mammogram (diagnostic) – unilateral | $28.05 | $16.90 |
| X185 | Mammogram (diagnostic) – bilateral | $37.15 | $27.00 |
| X194 | Additional coned views with or without magnification (limit of two per breast) per film | $5.95 | $5.20 |
| X166 | Examination using portable machine ($72.20 fee from Schedule of Facility Fees) | n/a | n/a |
| J127 | Vascular ultrasound breast – scan B-mode per breast | $23.70 | $13.10 |
| J037 | Lymphangiogram – Mammary ductography | n/a | $70.35 |
| E475 | Portable ultrasound - only eligible for payment when personally rendered by a specialist in diagnostic radiology (33) in an area of a hospital outside of the diagnostic imaging department | n/a | $25.00 |
| X446 | MRI breast (unilateral/bilateral) – multi-slice sequence | n/a | $73.35 |
| X447 | MRI breast (unilateral/bilateral) – repeat (another plane, different pulse sequence - to a maximum of 3 repeats). | n/a | $36.70 |
| X192 | CT – Mammary ductography | $25.05 | $10.65 |
| X121 | Stereotactic core breast biopsy | n/a | $83.15 |
| X201[c] | Breast biopsy specimen x-ray, per specimen | $5.95 | $5.20 |
| X480 | MRI guidance of biopsy or lesion ablation, breast, unilateral | n/a | $285.00 |
| L864 | SURGICAL PATHOLOGY, LEVEL 4. Gross and microscopic examination of breast (biopsy, not requiring microscopic evaluation of surgical margin; reduction mammoplasty) | n/a | $48.65 |
| L865 | SURGICAL PATHOLOGY, LEVEL 5. Gross and microscopic examination of breast (partial or simple mastectomy; excision requiring microscopic evaluation of surgical margin) | n/a | $103.20 |
| L866 | SURGICAL PATHOLOGY, LEVEL 6. Gross and microscopic examination of breast (mastectomy with regional lymph nodes) | n/a | $181.65 |
| L805 | Aspiration biopsy e.g., lung, breast, thyroid, prostate | n/a | $81.20 |
| Z141 | Needle biopsy (breast) – one or more | n/a | $37.20 |
| Z143 | Needle biopsy - large core breast biopsy - (14 gauge or larger bore needle) | n/a | $132.75 |
| R107 | Tumour or tissue for diagnostic biopsy and/or treatment, e.g., carcinoma, fibroadenoma or fibrocystic disease (single or multiple - same breast) | Asst*6<br>Anae*6 | $169.95 |
| J149 | Ultrasonic guidance of biopsy, aspiration, amniocentesis or drainage procedures (1 physician only) | $47.30 | $36.85 |

| | | | |
|---|---|---|---|
| E525 | Tumour or tissue for diagnostic biopsy and/or treatment, e.g., carcinoma, fibroadenoma or fibrocystic disease (single or multiple - same breast) – after localization with mammographic wire or radioactive seeds | n/a | $48.05 |
| X169 | DIAGNOSTIC RADIOLOGY-LAMINOGRAM,PLANIGRAM,TOMOGRAM (default use Ontario cost) | n/a | $51.25 |
| A225 | Consultation | n/a | $167.35 |
| A220 | Genetics - Special genetic consultation | n/a | $300.70 |
| A223 | Genetics - Extended special genetic consultation | n/a | $401.30 |
| A325 | Limited consultation | n/a | $105.25 |
| A226 | Repeat consultation | n/a | $105.25 |
| A221 | Genetic minor assessment | n/a | $38.05 |
| K016 | Genetic assessment, patient or family, direct contact, per | n/a | $74.05 |
| K222 | Genetic care per ½ hr | n/a | $75.75 |
| K223 | Clinical interpretation | n/a | $38.20 |
| K229 | Complex genetic test interpretation | n/a | $65.85 |
| K044 | Genetic counselling on behalf of a third party | n/a | $62.75 |

[a]If X194 is billed on the same day as a screening mammogram code (i.e., X172 or X178) then cost added to screening mammogram costs. If X194 is billed without a screening mammogram code then cost added to diagnostic mammogram costs.
[b]If X166 is billed on the same day as a screening mammogram code (i.e., X172 or X178) then cost added to screening mammogram costs. If X166 is billed without a screening mammogram code then cost added to diagnostic mammogram costs.
[c]If X201 is billed only and there are no other biopsy codes billed during follow-up (i.e., E525, J149, L805, L864-L866, R107, X121, X480, Z141, or Z143) then exclude X201 from diagnostic biopsy costs.

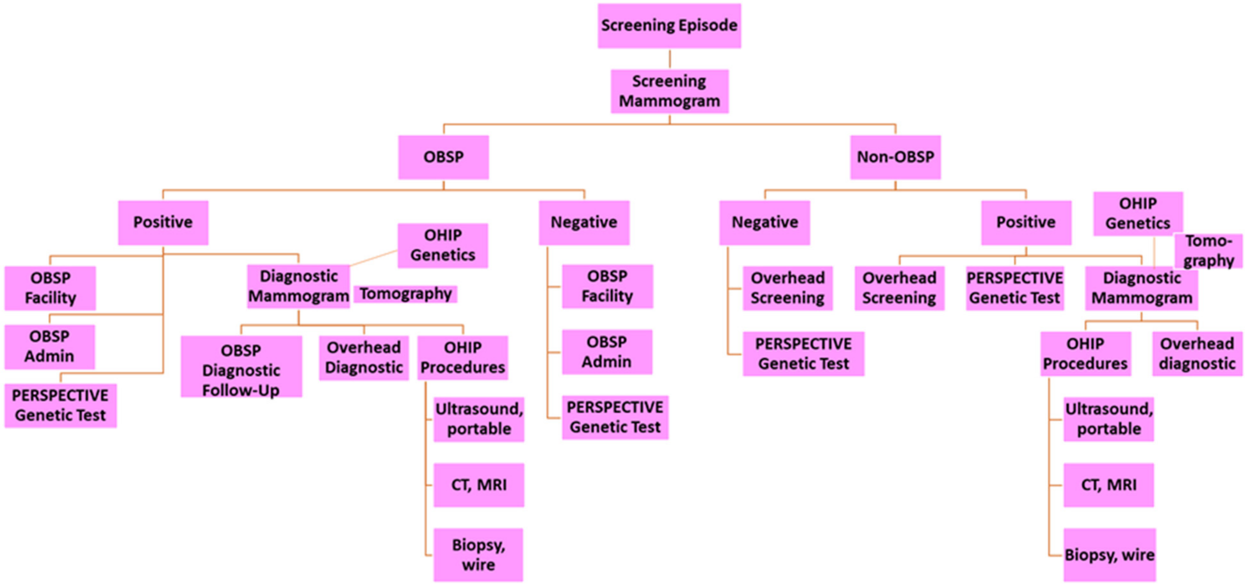


**Figure S1.** Breast cancer screening schematic.

**Table S3.** Ontario specific cost assumptions.

| OBSP | COST |
|---|---|
| OBSP facility cost (IHF=$15.97, hospital=$21.57) based on database flag for screening mammogram location | IHF= $15.97<br>Hospital= $21.57 |
| OBSP administrative cost (M. Pistilli, personal communication, January 10, 2022) | $17.52 |
| OBSP diagnostic follow-up cost for those women who had any OHIP diagnostic procedures | $100.00 |
| NON-OBSP | |
| Non-OBSP institution/overhead cost | $51.22 |

**Table S4.** Quebec specific unit costs.

| CODE | DESCRIPTION | QC Tech Cost 2021 | QC Prof Cost 2021 |
|---|---|---|---|
| 08103 | Mammogram (screening) – unilateral | $24.70 | $9.20 |
| 08079 | Mammogram (screening) – bilateral | $40.55 | $18.45 |
| X194 | Additional coned views with or without magnification (limit of two per breast) per film USE ONTARIO COSTS | $5.95 | $5.20 |
| 08146 | Examination using portable machine (mobile unit) | Assume $0 | $18.35 |
| 08048 | Mammogram (diagnostic) – unilateral | $26.80 | $10.05 |
| 08049 | Mammogram (diagnostic) – bilateral | $40.55 | $19.85 |
| X194 | Additional coned views with or without magnification (limit of two per breast) per film USE ONTARIO COSTS | $5.95 | $5.20 |
| 08146 | Examination using portable machine | Assume $0 | $18.35 |
| J127 | Vascular ultrasound breast – scan B-mode per breast USE ONTARIO COSTS | $23.70 | $13.10 |
| 60705 | Lymphangiogram – Mammary ductography USE QC BILATERIAL SURFACE ULTRASOUND | $67.95 | $70.20 |
| E475 | Portable ultrasound - only eligible for payment when personally rendered by a specialist in diagnostic radiology (33) in an area of a hospital outside of the diagnostic imaging department USE ONTARIO COST | $25.00 | Assume $0 |
| 08590 | MRI breast (unilateral/bilateral) – multi-slice sequence | Assume $0 | $151.70 |
| X447 | MRI breast (unilateral/bilateral) – repeat USE ONTARIO COST | Assume $0 | $36.70 |
| X192 | CT – Mammary ductography USE ONTARIO COST | Assume $0 | $35.70 |
| 20068 | Stereotactic core breast biopsy (BREAST BIOPSY WITH SUCTION-ASSISTED MACROBIOPSY DEVICE) | Assume $0 | $166,25 |
| X201 | Breast biopsy specimen x-ray, per specimen DEFAULT USED ONTARIO COST | $5.95 | $5.20 |
| 00852 | MRI guidance of biopsy or lesion ablation breast (UNILATERAL HARPOON UNDER U/S OR STEREOTACTIC GUIDANCE) | Assume $0 | $121.70 |
| L864 | SURGICAL PATHOLOGY, LEVEL 4. Gross and microscopic examination of breast (biopsy, not requiring microscopic evaluation of surgical margin; reduction mammoplasty) DEFAULT USED ONTARIO COST | Assume $0 | $48.65 |
| L865 | SURGICAL PATHOLOGY, LEVEL 5. Gross and microscopic examination of breast (partial or simple mastectomy; excision requiring microscopic evaluation of surgical margin) DEFAULT USED ONTARIO COST | Assume $0 | $103.20 |
| L866 | SURGICAL PATHOLOGY, LEVEL 6. Gross and microscopic examination of breast (mastectomy with regional lymph nodes) DEFAULT USED ONTARIO COST | Assume $0 | $181.65 |
| 00847 | Aspiration biopsy e.g. lung, breast, thyroid, prostate (BREAST CYST PUNCTURE W/ASPIRATION) | Assume $0 | $18.70 |
| Z141 | Needle biopsy (breast) – one or more USE ONTARIO COST | Assume $0 | $37.20 |
| 00849 | Needle biopsy - large core breast biopsy - (14 gauge or larger bore needle) | Assume $0 | $128.45 |

| | | | |
|---|---|---|---|
| 01201 | Tumour or tissue for diagnostic biopsy and/or treatment, e.g. carcinoma, fibroadenoma or fibrocystic disease (single or multiple - same breast) (BIOPSY FOR SINGLE/MULTIPLE BENIGN/MALIGNANT LESION OF BREAST) | Assume $0 | $212.90 |
| J149 | Ultrasonic guidance of biopsy, aspiration, amniocentesis or drainage procedures (1 physician only) USE ONTARIO COST | $47.30 | $36.85 |
| 20029 | Tumour or tissue for diagnostic biopsy and/or treatment, e.g. carcinoma, fibroadenoma or fibrocystic disease (single or multiple - same breast) – after localization with mammographic wire or radioactive seeds (INSTALLATION OF MARKER GUIDE) | Assume $0 | $29.15 |
| n/a | OBSP facility cost - Code 08081 | $7.50 | n/a |
| n/a | OBSP administrative cost | $17.52 | n/a |
| n/a | OBSP diagnostic follow-up cost - Code 08144 | $59.80 | n/a |
| n/a | Overhead cost | $51.22 | n/a |
| X169 | DIAGNOSTIC RADIOLOGY-LAMINOGRAM,PLANIGRAM,TOMOGRAM (default use Ontario cost) | n/a | $51.25 |
| Z141 | Needle biopsy (breast) – one or more (DEFAULT USE ONTARIO COST) | n/a | $37.20 |
| A221 | Genetic minor assessment (DEFAULT USE ONTARIO COST) | n/a | $38.05 |
| K016 | Genetic assessment, patient or family, direct contact, per (USED QC CODE 9164 CONTROL VISIT) | n/a | $67.60 |
| K229 | Complex genetic test interpretation (USED QC CODE 9164 CONTROL VISIT) | n/a | $67.60 |
| K044 | Genetic counselling on behalf of a third party (USED QC CODE 9164 CONTROL VISIT) | n/a | $67.60 |
